# The Relationship between Internalising Symptom Development and Academic Attainment in Early Adolescence

**DOI:** 10.1371/journal.pone.0116821

**Published:** 2015-01-21

**Authors:** Praveetha Patalay, Jessica Deighton, Peter Fonagy, Miranda Wolpert

**Affiliations:** 1 Evidence Based Practice Unit (EBPU), University College London and the Anna Freud Centre, 21 Maresfield Gardens, London,United Kingdom; 2 University College London, Gower Street, London, United Kingdom; Univ of Toledo, UNITED STATES

## Abstract

Evidence for the longitudinal associations between internalising symptom development and academic attainment is sparse and results from existing studies are largely inconclusive. The approaches that have been used in existing studies examining this relationship have in common the limitation of grouping together all individuals in the sample which makes the assumption that the relationship between time, symptoms and attainment across all individuals is the same. The current study aimed to use heterogeneous trajectories of symptom development to examine the longitudinal associations between internalising symptom development and change in academic attainment over a three years period in early adolescence, a key period for internalising symptom development. Internalising symptoms were assessed for 3 consecutive years in a cohort from age 11–14 years (n = 2647, mean age at T1 = 11.7 years). National standardised test scores prior to the first wave and subsequent to the last wave were used as measures of academic attainment. Heterogeneous symptom development trajectories were identified using *latent class growth analysis* and socio-demographic correlates, such as gender, SES and ethnicity, of the different trajectory groupings were investigated. Derived trajectory groupings were examined as predictors of subsequent academic attainment, controlling for prior attainment. Results demonstrate that symptom trajectories differentially predicted change in academic attainment with increasing trajectories associated with significantly worse academic outcomes when compared to pupils with low levels of symptoms in all waves. Hence, a trajectory based approach provides a more nuanced breakdown of complexities in symptom development and their differential relationships with academic outcomes and in doing so helps clarify the longitudinal relationship between these two key domains of functioning in early adolescence.

## Introduction

Internalising symptoms are the most prevalent of mental health problems in childhood and adolescence [1], and in adulthood are one of the largest causes of health burden and years of life lost [2]. Symptoms in childhood and adolescence are a strong predictor of developing a diagnosis in adulthood [3]. On the other hand, educational outcomes in childhood and adolescence are a predictor of many childhood and adult outcomes including occupation, earnings, health and mortality [4]. Hence, both internalising symptoms and academic attainment not only have important implications for concurrent functioning in youth, but also predict lifelong economic and social outcomes.

Interest in the associations between key domains of development and functioning, such as mental health and academic attainment, is theoretically based on the assumption that different domains are linked developmentally [5]. Positive development or success in one domain is expected to provide scaffolding for positive development in the same and other domains; and conversely, negative development or deficits in one domain can result in negative development in the same and other domains [6]. Therefore, understanding the longitudinal associations between internalising symptoms and academic attainment over childhood and adolescence are of interest both from a developmental perspective and also from a perspective of intervention and school based support. Better understanding these relationships in community based school samples can contribute to the debate about early intervention and school-based support for mental health difficulties, and the need for greater integration between prevention and educational policy [7].

Cross-sectional associations between internalising symptoms and academic attainment have been established, with higher levels of symptoms being associated with greater academic problems [8, 9]. However, evidence for the longitudinal associations between internalising symptoms and academic attainment is sparse and results from existing studies are largely inconclusive [10]. Methodologies used to explore these longitudinal associations have included: early symptoms predicting later outcomes [11, 12], aggregate symptoms predicting change in educational outcomes [13], and more complex approaches such as cascade modelling [10, 14]. Cole et al [13] using an aggregate symptoms predicting subsequent attainment approach found no significant effects of internalising symptom levels in a large sample of American adolescents. Similarly, studies using cascade analysis have found little or no links between earlier internalising symptoms with later academic competence [14]. Studies examining this relationship in large datasets across different countries (Canada, US, UK) did not find significant links between emotional symptoms to later educational attainment [11, 15]. Conversely, links have been found between adolescent levels of depression and education attained at age 21[16], and negative impact of internalising symptoms in early adolescence on subsequent attainment within the same academic year [17].The difficulty in establishing longitudinal associations in some part might be attributed to the complex nature of internalising symptom development [18]. However, the approaches that have been used in existing studies examining this relationship have in common the limitation of grouping together all individuals in the sample which makes the assumption that the relationship between time, symptoms and attainment across all individuals is the same [19]. The authors did not identify any study that has assessed the differential impact of heterogeneous symptom trajectories on educational outcomes.

Identifying and studying the correlates of heterogeneous trajectories of internalising symptoms has gained popularity in the last decade as it allows partitioning of the effects of variables and time on different individuals [19]. Studies have identified and examined the predictors associated with trajectories of internalising symptoms [20, 21], and also more specifically of depression [22], and anxiety [23–25]. Groupings of symptom development achieved by this method probabilistically identify individuals as belonging to a certain trajectory grouping. Although this does not mean all individuals in a group have exactly the same trajectory, it does indicate that their trajectories are similar to each other and different from individuals identified as having other trajectories [26]. Hence, the correlates and predictors of the different trajectory groups can also be subsequently identified. Existing investigations have contributed greatly to the study of the development of internalising psychopathology in childhood and adolescence; however, the focus has mainly been on recognising long term trajectories and evaluating their associated variables and risk factors. These studies have in common: 1) focus on long-term pathways (e.g. from 4–18years [22]), 2) use of proxy reported symptoms and 3) a lack of focus on the predictive capacity of different symptom development trajectories on educational and other outcomes. The only educational variable that has been explored using a trajectory based approach is highest level of education attained by adulthood [27]. There is no research that has focussed on the shorter term impacts of increases and decreases in symptomology on academic attainment.

Longitudinal investigations indicate that internalising symptoms become more prevalent around puberty and peak at around ages 13–15 years and then prevalence decreases going into late adolescence/early adulthood [28, 29]. Hence, the period in early adolescence from age 11–14 years is characterised by higher levels of symptom development and symptoms peak in middle adolescence around age 13–15 years [30, 31]. The correlates and impact on other domains such as academic outcomes of symptoms development at this particularly vulnerable stage has not been the focus of detailed study.

The current study aims to investigate the impact of different symptom development trajectories from 11–14 years on subsequent academic outcomes when controlling for prior academic performance. To do so, we combine the two lines of research that are discussed above and utilise a heterogeneous trajectories methodology to investigate the longitudinal associations between internalising symptoms and academic attainment. We aim to do this by first identifying empirically derived heterogeneous trajectories in self-reported symptoms over three waves from age 11–14 years. Once identified, the socio-demographic correlates of different symptom trajectories will be explored to identify unique associations between risk factors and different symptom pathways. Subsequently, the impact of the different symptom trajectories on subsequent academic attainment will be analysed while controlling for prior attainment so as to reflect their associations with relative gains or losses made in learning during the same three year period. This approach has the potential to offer a clearer, more nuanced picture of the impact that different symptom development pathways of individuals can have on their academic learning during early adolescence. Based on developmental ecosystem and dynamic system frameworks [5, 32] and theories of developmental competence [6], which predict that positive development in one area encourages positive development in the same and other domains (and vice versa in the case of negative development), we hypothesise that increasing symptoms will be associated negatively with subsequent academic attainment.

## Method

### Design

**Figure 1:**
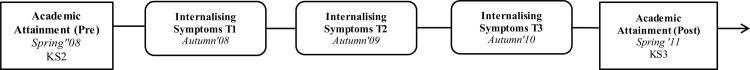
Study design illustrating when data for key variables—educational attainment and internalising symptoms, were collected.

Fig. 1 outlines the time-frames of the study and data collection. Internalising symptoms were assessed in the first term of schooling (which is during autumn in England) every consecutive year for three years. Academic attainment scores were taken from national standardised tests at the end of a Key Stage (KS) in England which approximately correspond to age 11(KS2) and age 14 (KS3) (see www.education.gov.uk/ for detailed descriptions of KSs). As a result all participants had KS2 scores prior to the start of the study and KS3 scores subsequent to the three waves of internalising symptoms assessment (see Fig. 1). Hence, the impact of symptom development during the three waves on relative change (gains or losses) in national standardised tests of academic attainment can be examined.

### Participants

Data from a 3-year longitudinal study of mental health in schools in England were utilised in this study. Greater details of the wider research project are published elsewhere [33]. Data were collected at yearly intervals from 37 secondary schools who participated in all three waves of the study. In wave 1, 5087 pupils participated in the survey. Due to the requirement of a minimum of three time points to estimate person-centred growth trajectories [34, 35] 2,647 pupils (52%) who had participated in all three years were included in analysis. Attrition was mainly due to absenteeism and entire form groups within some schools being unable to participate in some waves.

Participants were 11.71 years (SD = .29) at wave 1 and 54.4% of participants were female. Free school meal (FSM) eligibility was higher than national levels at 17.1% (vs.11.9% nationally [36]). The majority of participants were classified as White (73.6%) followed by Asian (17.5%), Black (4.9%), mixed (3%) and other (1.1%). 7.1% of the participants were classified as having special educational needs (SEN). Academic attainment scores in national standardised tests (M = 27.7, SD = 4.19) was identical to the national average [36].

Attrition analysis was carried out to assess whether individuals lost to follow-up waves were different from the final sample. There were no differences in gender proportions (*χ* ^2^ = .01, p = .93) and SES (*χ* ^2^ = 3.10, p = .08). The final sample had lower proportions of ethnic minorities (*χ* ^2^ = 4.47, p<.05) and students with SEN (*χ* ^2^ = 4.52, p<.05) when compared to the sample at Time 1. Mean internalising scores at time 1 were lower for the final sample (t = 2.34, p<.05) and prior academic attainment was higher in the final sample (t = 3.79, p<.001).

In summary, the final sample analysed in this study is representative of pupils nationally except for deprivation, as the study sample has a higher proportion of deprived pupils compared to national levels. However, analysis of attrition indicates that pupils lost in follow-up waves were significantly more likely to have SEN, belong to ethnic minority groups, have greater internalising symptoms and lower prior attainment scores.

### Procedure

Computer-based surveys were completed by pupils within the normal school day with support from their class teachers. Teachers were given standardised information to read out to participants which included information about the study, an explanation regarding the confidentiality of their responses and their right to decline to participate and drop out at any time. Data on socio-demographic characteristics such as gender, deprivation, ethnicity and age were obtained from the national pupil database, a centrally collated database that holds all education related data on all school students in England. Academic attainment scores in national standardised tests were also obtained from this database.

#### Ethics statement

Ethics permission for the study and data collection was granted by the research ethics committee of University College London (REF: 1530/001). Consent for collecting the data was sought from parents of participants and from the participants themselves prior to each wave of data collection. Parents/carers received information sheets and consent forms prior to each time point of assessment and returned the completed consent form if they wanted to opt their child out of the research. Participants themselves read information sheets, alongside the information sheet being explained to them (as outlined above in procedure), and proceeded to participate in the study with their consent recorded on the computer system.

### Measures

#### Internalising symptoms

Internalising symptoms were measured using the *Emotional Difficulties* scale of the Me and My School questionnaire [37, 38], which is a 10-item self-report scale (e.g. ‘I feel lonely’, ‘I worry a lot’) with three response options: never, sometimes, always. The answers to the items are summed to create a total emotional difficulties score, higher scores indicating more difficulties. The measure has good content validity, internal reliability, discriminant capacity and construct validity and items do not operate differently across sub-groups based on SEN, deprivation and English as additional language [37, 38].The scale has an at-risk cut-off score of 10 (10–11 borderline, >12 clinical) [37]). Cronbach’s ranged from .77–.80 at each time point indicating good internal reliability.

#### Academic Attainment

National standardised test results (referred to as Key Stages [KS] in England), averaged across English, mathematics and science were used as a measure of attainment. Hence, the KS2 levels (M = 4.16, SD = .66, range 1–5) and KS3 levels (M = 5.54, SD = .95, range 1.67–8) were used as measures of attainment pre and post the three waves respectively.

### Correlates

#### Gender

Participants reported on their gender and this information was cross-referenced with school help information and the NPD to create a gender variable. In all analysis males were coded ‘0’ and females ‘1’.

#### Ethnicity

Ethnicity information was divided into the broad categories of White, Asian, Black, mixed and other (other consisted of participants belonging to groups with very low proportion [e.g. Gypsy, 0.1%], refusing information, or their ethnicity code recorded as unclassified).

#### Socio-economic status (SES)

SES was measured by free school mean eligibility (FSM), which is a widely used proxy for deprivation in school-based research [39]. The binary variable was coded ‘0’ for not eligible and ‘1’ for participants who were eligible for free school meals.

#### Age

Age was estimated to the month from month and year of birth data available for each participant at wave 1 of data collection. As chronological age was accounted for by the time-points year on year, the age variable in the models represents age within the cohort at any time-point, representing relative age within the cohort. At time 1, mean age was 11.71 years (SD = 0.29) with 99.9% of the sample ranging from 11.25 years to 12.4 years.

#### Special Educational Needs

SEN status was recorded from the National Pupil Database (NPD) and students were included as having SEN if they had either a SEN statement or school provision to support SEN (in NPD referred to as Statemented and School Action Plus). Individuals were assigned ‘1’ if they had either statement or school action plus and ‘0’ if not.

### Analytic Strategy

The current study aimed to use symptom development in the three waves between attainment measures as a predictor of change in academic attainment over that corresponding period of time. Recognising that symptom development is heterogeneous in a sample, we decided to use empirically derived trajectories to summarise different developmental pathways over three waves. Hence analyses were conducted in multiple steps. The preliminary analysis (Stage 1) involved identifying heterogeneous developmental trajectories of internalising symptoms and investigating the socio-demographic and educational predictors of the derived symptom trajectories. Subsequently, the trajectory groupings were utilised as predictors to examine the associations of different trajectories with change in academic attainment (Stage 2).

#### Stage 1: Identifying heterogeneous developmental trajectories and their predictors

Latent class growth analysis (LGCA), a semi-parametric technique which identifies sub-groups of individuals following a similar pattern over time[40], was conducted in Mplus7 [41] to estimate empirically derived trajectory models and identify a *k*-trajectory model that had good fit criteria, parsimony and theoretical interpretability. Criteria used to assess and select a *k*-trajectory model for further analysis included model fit, neatness of classification and interpretability [42]. Model selection was based on comparing log likelihood estimates of *k*-trajectory model with *k-1* trajectory model using the Lo-Mendell-Rubin likelihood ratio test (LMR-LRT). Model fit was estimated using the sample-adjusted Bayesian information criterion (BIC), neatness of classification was assessed using entropy and posterior probabilities and interpretability was assessed based on known theoretical models, clinical usefulness and proportions in identified groups [42].

To explore the correlates and risk factors associated with the heterogeneous trajectories identified, multinomial logistic regressions were conducted were conducted in STATA12 [43] comparing each trajectory to the reference group (trajectory with the largest proportion). Gender, ethnicity, deprivation, age, SEN and prior academic attainment were included as predictors in the analysis due to their established associations with internalising symptoms [1]. Relative risk ratios (RR) that represent the probability of having a certain trajectory when compared to the reference group for the predictor of interest were estimated [44]. A RR greater than 1 indicates that the risk is increased for the predictor category/unit change in predictor and, inversely, RRs less than 1 indicate reduced risk [44].

#### Stage 2: Predicting academic attainment

The main analysis aimed to examine the associations between the derived trajectories and change in academic attainment over the three waves by including trajectory groups in the model predicting attainment post the final wave while controlling for attainment prior to the initial wave. As school-level variation in attainment was high (>20%), multi-level modelling was utilised to account for nesting of participants within schools. The multi-level models were computed using both aggregated symptom scores over three waves and the derived trajectories as predictors to allow comparison of the predictive utility of the trajectory based approach. Models were computed in STATA12 [43] using the derived trajectories as categorical predictors by creating a dummy variable where, like in the previous stage, the group with the highest proportion was the reference group. Effect sizes for main effects were computed by dividing the beta estimate for main effect by the average of the square root of the variance estimate at both time-points [45].

## Results

### Stage 1: Identifying heterogeneous developmental trajectories and their predictors

**Figure 2:**
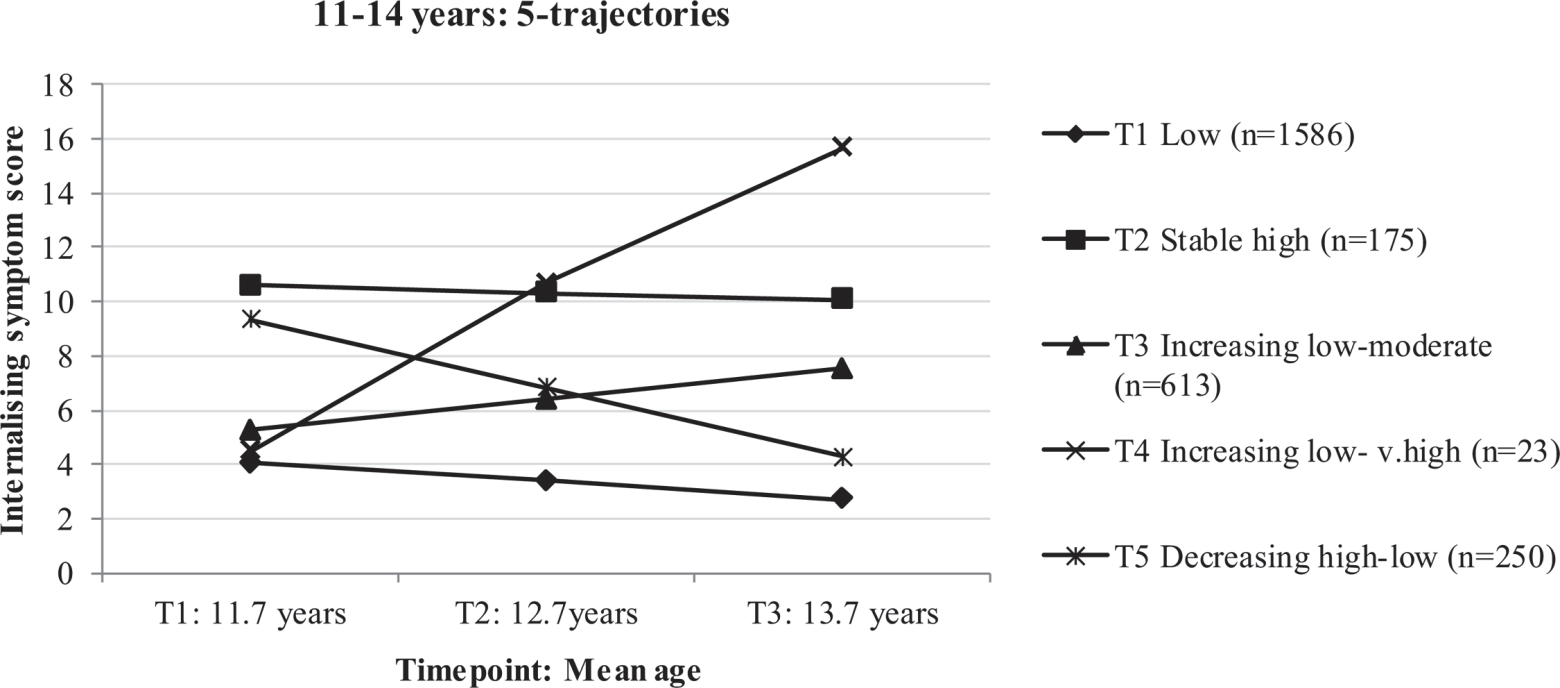
Heterogeneous developmental trajectories of internalising symptoms from age 11 to 14 years.

Two- to seven-trajectory models were estimated to select a k-trajectory model that summarised symptom development over the three waves. Log-likelihood differences, LMR-LRT, for each of the models as compared to the previous model were as follows: 2-trajectory = 180.92. 3-trajectory = 126.16, 4-trajectory = 71.68, 5-trajectory = 118.44, 6-trajectory = 26.15, and 7-trajectory = 24.42.The large drop in improvement in model from the 5- to 6- trajectory model is observed, which also represents a non-significant improvement of model in these data indicating the 6-trajectory model was not a better fit to the data than the 5-trajectory model. The 5-trajectory model also demonstrated sufficient neatness of classification (entropy = .73) and posterior probabilities (.72–.88). Hence, the model with 5-trajectories was selected for further exploration (presented in Fig. 2).

Descriptive analysis of the trajectories indicated there were two trajectories of significantly increasing symptoms over time (23.2% and .9%), representing almost a quarter of participants who developed internalising symptoms between ages 11 and 14 years. Symptoms significantly decreased from high in 9.4% of the sample and a large majority (almost 60%) had low internalising symptoms over the three waves. Table 1 presents the sample breakdown and intercept and slope co-efficients for the *5-trajectories*. School-level variation in trajectories was small (ICC = .01) and hence nesting of students within schools was not statistically accounted for in the analysis below: exploring the correlates of trajectories [46].

**Table 1:** Sample breakdown and intercept and slope co-efficients by trajectory group.

**Trajectory**	**N (%)**	**Gender% (Female)**	**FSM% (Yes)**	**Age M (SD)**	**SEN% (Yes)**	**Academic Attainment M (SD)**	**Intercept**	**Slope (p)**
T1 *Low*	1586(59.9)	48.9	14.9	11.71(.29)	5.5	4.20(.64)	4.04	−.66***
T2 *Stable high*	175(6.6)	74.9	20.7	11.71(.29)	13.1	3.98(.78)	10.6	−.27(.11)
T3 *Increasing low-moderate*	613(23.2)	62.6	19.9	11.71(.30)	7.8	4.14(.64)	5.27	1.13***
T4 *Increasing low-high*	23(.9)	26.1	17.4	11.67(.26)	13	4.23(.79)	4.47	5.6***
T5 *Decreasing high-low*	250(9.4)	57.2	20.9	11.69(.30)	10.4	4.05(.70)	9.34	−2.52***
Overall sample	2637	54.4	17.1	11.71(.29)	7.1	4.16(.66)	5.53	−.4***

***p< .001.

Based on the previous analysis, the trajectory with the largest proportion: the low symptom trajectory group was taken as the reference group for the multinomial regression model. Gender, ethnicity, deprivation, age, SEN and prior academic attainment were included as correlates in the analysis and results are presented in Table 2.

**Table 2:** Multinomial logistic regression examining correlates in the five-trajectory model.

**Predictors**	**T1**	**T2**			**T3**			**T4**			**T5**		
	***Low***	***Stable high***			***Increasing low-moderate***			***Increasing low-high***			***Decreasing high-low***		
		**RR(SE)**	**95% CI**		**RR(SE)**	**95% CI**		**RR(SE)**	**95% CI**		**RR(SE)**	**95% CI**	
Gender (Female)	Reference group	3.43***(.66)	2.35	5.01	1.79***(.18)	1.46	2.18	.36*(.18)	.14	.94	1.51**(.22)	1.13	2.00
Ethnicity-Asian		.63*(.15)	.39	1.02	.88(.12)	.67	1.14	2.02(1.09)	.70	5.82	1.07(.19)	.75	1.52
Ethnicity-Black		1.55(.48)	.84	2.83	.95(.22)	.60	1.50	1.72(1.83)	.22	13.77	.92(.31)	.47	1.78
Ethnicity-Mixed		1.27(.58)	.52	3.10	1.10(.32)	.63	1.93	4.52^(3.55)	.97	21.04	.87(.39)	.36	2.10
Ethnicity-Other		1.25(.97)	.28	5.68	1.49(.63)	.65	3.42	.00(.004)	0	-	.00(.001)	0	-
FSM (Yes)		1.27(.27)	.83	1.92	1.28^(.17)	.99	1.66	.94(.60)	.27	3.31	1.34(.25)	.94	1.91
SEN (Yes)		2.06**(.61)	1.15	3.69	1.54*(.32)	1.03	2.31	2.43(1.96)	.82	12.58	1.65^(.44)	.98	2.77
Age		.97(.28)	.56	1.71	.96(.16)	.69	1.34	.53(.40)	.12	2.13	.82(.20)	.51	1.31
KS2		.68**(.09)	.53	.87	.94(.08)	.80	1.10	1.75(.70)	.79	3.85	.79*(.09)	.64	.98

***p< .001

**p< .01

*p< .05

^p< .10.

As can be seen from Table 2, being female predicted higher risk of having the higher symptom trajectories, except the increasing low-high trajectory where males were significantly more likely to belong to this trajectory group. Asian ethnicity was associated with a smaller probability of being in the stable high group (RR = .63, p<.05). SEN significantly predicted higher risk of having stable high and increasing trajectories. Prior academic attainment significantly predicted the stable high and decreasing trajectories, with higher scores indicating lower risk of having stable high symptoms (RR = .68, p<.01) and decreasing symptoms (RR = .79, p<.05).

### Stage 2: Predicting academic attainment

Results of the multi-level regression model are presented in Table 3; the first model (A) controls for prior attainment and socio-demographic and educational predictors, the second model (B) is model A plus aggregated symptom scores and the third model (C) is model A with trajectories added in as categorical predictors; the reference category being the low decreasing symptoms trajectory.

**Table 3:** Multi-level models predicting change in academic attainment.

**Parameter Estimates (Outcome: KS3)**	**Model A**	**Model B**	**Model C**
	**Estimate (SE)**	**Estimate (SE)**	**Estimate (SE)**
*Fixed Effects*			
Intercept	1.15** (.41)	1.26** (.41)	1.20** (.41)
Prior Attainment:KS2	1.12*** (.02)	1.12*** (.02)	1.12*** (.02)
Gender (Female)	.06** (.02)	.07*** (.02)	.07** (.02)
FSM (Yes)	−.13*** (.03)	−.12*** (.03)	−.12*** (.03)
Ethnicity (Asian)	.04 (.04)	−.16*** (.04)	−.16*** (.04)
Ethnicity (Black)	.06 (.06)	.03 (.04)	.03 (.04)
Ethnicity (Mixed)	.06 (.06)	.06 (.06)	.06 (.05)
Ethnicity (Other)	.21* (.10)	.06 (.06)	.06 (.06)
SEN (Yes)	−.17*** (.04)	.20* (.10)	.20* (.10)
Age	−.02 (.03)	−.03 (.03)	−.03 (.04)
Aggregated symptoms		−.01*** (.00)	
T2 (Stable high)			−.06 (.04)
T3 (Increasing low-moderate)			−.05* (.025)
T4 (Increasing low-high)			−.18^ (.11)
T5 (Decreasing high-low)			−.05 (.04)
*Variance Components*			
Residual variance	.50 (.01)	.50 (.01)	.50 (.01)
School-level	.25 (.04)	.25 (.04)	.25 (.04)

***p< .001

**p< .01

*p< .05

^p< .10.

Model B indicates that aggregated symptoms significantly predict change in attainment scores, with higher problems indicating negative gain in attainment. The co-efficient of change is low (β = −.01, p<.001, ES = .01). Looking at the model in which different trajectories predict scores (model C), when compared to the low symptom trajectory pupils with increasing trajectories demonstrated significant or nearly significant negative co-efficients of change in attainment (increasing low-moderate β = −.05, p<.05, ES = .07; increasing low-high β = −.18, p<.10, ES = .20). The remaining two trajectory groups, stable high and decreasing symptom groups did not significantly predict worse change in attainment compared to the reference group (ES = .06 and .07).

## Discussion

The current study aimed to utilise a trajectory based approach to better understand the association between development of internalising symptoms and changes in academic attainment during early adolescence, a critical period of internalising symptom development. Analyses were conducted in multiple stages to first identify empirically derived trajectories of symptoms, assess the predictors that are associated with them and subsequently use the derived trajectories in a model predicting subsequent attainment after controlling for prior academic attainment.

Results from the first stage of analysis support existing person-centred studies by demonstrating the relevance of using heterogeneous trajectories that capture different symptom development pathways. The current study contributes to existing explorations of developmental trajectories as it focused on shorter term trajectories based on self-reported symptoms. As would be expected from the known prevalence of disorder in the community [1], the largest proportions of individuals were in the group with low levels of symptoms at all-time points (59%). More than 600 (24.1%) individuals had significant increases in symptoms from age 11–14 years which supports epidemiological findings that emotional problems increase in early adolescence and potentially peak in adolescence around age 13–15 years [47].

In terms of socio-demographic risk factors associated with trajectory membership, being female was almost universally linked with higher symptom trajectories (i.e. all trajectories excluding low), with one notable exception where males demonstrated significantly greater risk of having the steep increase trajectory. Deprivation predicted higher risk of having increasing trajectories which is supported by established links between deprivation and internalising symptoms that have been found in cross-sectional studies [1].

The results of the main analyses indicate that developmental trajectories differentially predict changes in academic attainment over a similar period of time. In line with expectations from systems theories and the hypothesis of the present study, increasing symptom trajectories over the three years adversely impacted on academic performance post wave three, which indicates that increasing problems were associated with a significant decrease in performance while controlling for previous attainment. This is consistent with the hypothesis that developing difficulties in one area of functioning, such as mental health, can have knock-on effects on other domains of functioning.

Considering the inconsistent results of existing examinations of these longitudinal relationships (outlined in the introduction), these results provide a more definitive answer to questions regarding longitudinal impacts of developing internalising symptoms in early adolescence and at the same time provide a more nuanced picture of the educational risk associated with variations in symptom development in early adolescence. As discussed in the introduction, the reasons for the existing inconsistencies in results might be associated with the aggregation of symptom development over individuals, thereby reducing the known complexity that is present in these data with detrimental effects on the clarity of conclusions that can be drawn. The use of person-centred methodology in the current study, clarifies previously inconsistent results by providing a better breakdown of which individuals seem to experience negative academic outcomes as a function of internalising symptom development. The type of analysis used also allows us to look at these relationships comparatively based on differences in symptom development. The results indicate more clearly the difference in the impact on individuals with increasing symptoms when compared to individuals who do not experience increases in symptom development over the same time period allowing for an estimation of the amount of the negative impact on individuals with increasing symptoms. In terms of the change in score, in the English education system reaching a level 5 (from a possible range of 0–8) by Key stage 3 is the government recommended target [50], hence a fifth of a level represents a substantial amount of loss in attainment.

The additional nuances in this relationship that are gained by such an approach lead to interesting questions regarding the extent of the ramifications of adolescent symptom development on other outcomes, both short and long term. Future research, with appropriate data, can look at the longer term outcomes (academic and other) of these increases in symptoms in adolescence to verify whether negative impacts can be seen long term as well or whether the impact is limited to adolescence. Moreover, future research with appropriate data and methods might look to investigate cross domain ‘which drives which’ questions using more person-centred approaches.

### Strengths and Limitations

The use of a large community based sample that is broadly representative of the general population is a particular strength of this study. In terms of measures, contrary to most studies of these associations over time that use self- or teacher- reported attainment (e.g. [10, 13]), we use national standardised test scores as our measures of academic attainment which are relatively free from reporter bias when compared to concurrent self-reported attainment scores; hence minimising the risk of inflated associations between the two domains. Additionally, the use of standardised test scores permits the interpretation of results in a national context and supports future attempts at replication. Furthermore, the use of self-reported emotional difficulties by young people is briefly discussed here as another strength of the current study. Although it is widely accepted that the gold standard is to use multiple reporters of the child’s mental health status [51], it is usually not practical in large community based longitudinal studies. This has resulted in the majority of studies using proxy (most commonly maternal) reported symptoms to identify heterogeneous trajectories of symptoms (e.g. [20, 22, 52]). Given 1) the increasing focus on children’s perspective being important and necessary [53, 54], and 2) overwhelming evidence indicating low agreement between parent and child report (r = ∼.2 [55], and parent reported symptoms at a younger age are weak predictors of child self-reported symptoms [52]; studies using child self-reported symptoms are valuable as they redress this imbalance and might provide valuable insights that proxy-report studies cannot.

In terms of the participants, although the final sample is large and nationally representative, attrition must be noted as a limitation. Participants lost in follow-up waves were more likely to be deprived, have SEN, belong to ethnic minority groups and have lower prior academic attainment. Results from previous studies [1] and the present study, indicate that such individuals possess a greater risk of having higher levels of symptoms, and hence the proportions of children with high stable symptoms or increasing symptoms might be underestimated. A methodological drawback in deriving trajectories as shown in this paper is the small size of some trajectories, which consisted of individuals with a steep increase or decrease in symptoms. While these trajectories are clinically meaningful and represent the small proportion of children who experience these large changes in symptomatology, when they are used as predictors of outcomes their lack of sufficient power must be considered when comparing the results of different groups; for instance the steep increasing trajectory group did not significantly predict subsequent attainment although the regression co-efficient was larger than the others, and only demonstrated a trend of an effect. Suggested solutions for this power issue include using larger samples or focused studies with higher proportions of at-risk individuals [22, 56].

The results of the current study focus on the longitudinal association between internalising symptoms and academic attainment with limited reference to child, family or school characteristics that might be risk factors for either or both outcomes. Contextualising these relationships and testing shared risk hypothesis of factors that are associated with these relationships would be an important next step.

### Implications

While increasing internalising symptoms in adolescence is a widely occurring and well-established phenomenon with limited scope for complete prevention, the clarification of the negative impact on other domains stresses the need for support systems and interventions to help prevent this tumultuous stage of development affect other areas of adolescents’ functioning (such as attainment). Considering symptoms in adolescence are a pre-cursor to adult symptoms [3], there might be a case for beginning support and coping strategies in adolescence to prevent spilling over of impact on other areas of functioning, both at this age and later in adulthood.

Over the past few years the focus of educational reforms has been on curriculum goals that are more academic and skills-oriented, resulting in social and emotional components of education taking a back seat [57]. The results of the current study lend support to the arguments for prevention, early intervention and school-based support for mental health difficulties, and the need for greater integration between prevention and educational policy [7]. They also support the need for universal approaches that focus on prevention of problems and promotion of well-being [57] alongside reactive approaches after problems have arisen.
